# Prediction of Survival with Alternative Modeling Techniques Using Pseudo Values

**DOI:** 10.1371/journal.pone.0100234

**Published:** 2014-06-20

**Authors:** Tjeerd van der Ploeg, Frank Datema, Robert Baatenburg de Jong, Ewout W. Steyerberg

**Affiliations:** 1 Department of Science, Medical Center Alkmaar/Inholland University, Alkmaar, The Netherlands; 2 Department of Otorhinolaryngology and Head and Neck Surgery, Erasmus Medical Center, Rotterdam, The Netherlands; 3 Department of Public Health, Erasmus Medical Center, Rotterdam, The Netherlands; Wake Forest University Health Sciences, United States of America

## Abstract

**Background:**

The use of alternative modeling techniques for predicting patient survival is complicated by the fact that some alternative techniques cannot readily deal with censoring, which is essential for analyzing survival data. In the current study, we aimed to demonstrate that pseudo values enable statistically appropriate analyses of survival outcomes when used in seven alternative modeling techniques.

**Methods:**

In this case study, we analyzed survival of 1282 Dutch patients with newly diagnosed Head and Neck Squamous Cell Carcinoma (HNSCC) with conventional Kaplan-Meier and Cox regression analysis. We subsequently calculated pseudo values to reflect the individual survival patterns. We used these pseudo values to compare recursive partitioning (RPART), neural nets (NNET), logistic regression (LR) general linear models (GLM) and three variants of support vector machines (SVM) with respect to dichotomous 60-month survival, and continuous pseudo values at 60 months or estimated survival time. We used the area under the ROC curve (AUC) and the root of the mean squared error (RMSE) to compare the performance of these models using bootstrap validation.

**Results:**

Of a total of 1282 patients, 986 patients died during a median follow-up of 66 months (60-month survival: 52% [95% CI: 50%−55%]). The LR model had the highest optimism corrected AUC (0.791) to predict 60-month survival, followed by the SVM model with a linear kernel (AUC 0.787). The GLM model had the smallest optimism corrected RMSE when continuous pseudo values were considered for 60-month survival or the estimated survival time followed by SVM models with a linear kernel. The estimated importance of predictors varied substantially by the specific aspect of survival studied and modeling technique used.

**Conclusions:**

The use of pseudo values makes it readily possible to apply alternative modeling techniques to survival problems, to compare their performance and to search further for promising alternative modeling techniques to analyze survival time.

## Introduction

Predicting the survival probability of patients is important for various purposes in biomedical research, such as patient counselling, medical decision making, and benchmarking. The conventional analysis of survival problems mainly relies on Kaplan-Meier analysis and Cox regression modeling to predict the survival probability in relation to predictor variables [1], [2].

Alternative modeling techniques are available, such as support vector machines and artificial neural networks [3]–[5], which might possibly provide better predictions. For example, feed forward *neural networks* were already used in 1998 for the analysis of censored survival data [6]. In 2007, applications of random survival forests were described [7]. In 2009, prognostic indexes were compared using data mining techniques and Cox regression analysis in breast cancer data [8].

In 2000, Schwarzer and Vach [9] reviewed the use of artificial neural networks in medical research and found several problems. A major problem was that some of the alternative techniques did not deal adequately with censoring, which is essential for analyzing survival data. The conventional analysis of survival outcomes requires two variables: the status of the patient (e.g. dead or alive) and the time point at which this status is measured. In 2008, Klein et al. [10], [11] proposed to predict the survival at particular time points using pseudo values, which combine the variables status and time point in one outcome variable. The use of these pseudo values in generalized estimating equation modeling (GEE) using a log-minus-log link function leads to statistically appropriate analyses, which are in line with the results of Cox regression modeling.

In the current study, we aimed to study the use of pseudo values for analyses of survival outcomes with other modeling techniques, including support vector machines (SVM), neural networks (NNET), general linear models (GLM), recursive partitioning (RPART) and logistic regression (LR). To compare the performance, we applied these techniques and conventional regression analysis in the prediction of survival of 1282 Dutch patients with Head and Neck Squamous Cell Carcinoma (HNSCC), using predictors as described in earlier studies [12]–[14]. The survival of this particular population of newly diagnosed patients with HNSCC has already been studied by applying conventional Kaplan-Meier analysis, Cox regression and random survival forests (RSF) to 60-month survival and overall survival [15]–[17].

## Methods

### Patients and Data

We considered a cohort of 1371 patients with Head and Neck Squamous Cell Carcinoma (HNSCC) of the oral cavity, pharynx or larynx, diagnosed at Leiden University Medical Centre. The data were obtained from files used in an earlier study [16]. The same data had been used before to derive a prediction model based on the Cox regression modeling technique [15]. Predictors in this model included Tumor location, Age at diagnosis, Gender, T-N-M classification (T = the extent of the primary tumor, N = the absence or presence and extent of regional lymph node metastasis, M = the absence or presence of distant metastasis) and Prior malignancies. In 2010, Datema et al. [16], [17] published an updated model including comorbidity according to the Adult Comorbidity Evaluation, based on a 27-item comorbidity index (ACE27) [18]. In our study, we excluded patients for whom comorbidity was unknown, resulting in a total of 1282 patients**.**

### Outcome Variables

We defined three outcome variables related to patient survival:

- The 60-month survival (dichotomous, dead or alive, ignoring censoring before 60 months)
- The pseudo values at 60 months (continuous)
- The estimated survival time (continuous)

We focused on 60-month survival, since this is a common time point in cancer research. We subsequently calculated pseudo values for the time points 12, 24,…, 288, and 300 months to reflect the individual survival patterns of patients using the R-package “Pseudo”. The pseudo values form a new set of observations to allow for analysis as if we had time-to-event data without censoring [10], [11].

The estimated survival time was calculated as the sum of the pseudo values at these time points, because this sum reflects the area under the survival curve and can be interpreted as the mean survival time. The choice for a time interval of 12 months was motivated by the wish to have around 25 time intervals per subject for sufficient accuracy in estimating the survival time. File S1 (appendix 1) gives a more detailed description of the calculation and interpretation of the pseudo values and the estimated survival time. For univariate analysis of 60-month survival and overall survival we used Kaplan-Meier analysis and Cox regression analysis.

### Modeling Techniques

We considered the following modeling techniques: support vector machines (SVM), neural networks (NNET), recursive partitioning (RPART), general linear models (GLM) and logistic regression (LR), with their implementations as available in the software package R, version 2.14.1 [19]. The parameters of the various modelling techniques are presented in Table 1.

**Table 1:** Parameters required for the modeling techniques.

**Modeling technique**	**Parameters**
NNET	size and decay
RPART	cp-value
SVM LINEAR	cost and gamma
SVM POLYNOMIAL	cost, gamma and degree
SVM RADIAL	cost and gamma

File S2 (appendix 2) presents a more detailed description of the various modeling techniques and their parameters, based on previous literature [20]–[27].

### Tuning of the Modeling Techniques

Before applying a modeling technique, we tuned that technique by varying the parameters to create an optimal model fit. The optimal parameter setting was based on the smallest prediction error after 10-fold cross validation. The modeling technique SVM was tuned using a simultaneous grid search for the parameters cost and gamma when a radial or linear kernel was used and for the parameters cost, gamma and degree when a polynomial kernel was used. The modeling technique NNET was tuned using a simultaneous grid search for the parameter size, and the modeling technique RPART was tuned by varying the cp-value.

### Validation and Performance of the Modeling Techniques

For all models, internal validation was done by bootstrap resampling (200 bootstrap samples). From the original data set a bootstrap sample was drawn (randomly and with replacement). Then the modeling technique was tuned to create an optimal model fit for this bootstrap sample. With the optimal setting resulting from the tuning, we applied the modeling technique to the bootstrap sample and calculated the performance of the resulting model (bootstrap performance). We then applied the model to the original data base and calculated the performance (validated performance). This process was repeated 200 times. The 200 results were averaged to produce a single estimation of the bootstrap performance and the validated performance [28]. The difference of the mean bootstrap performance and the mean validated performance indicated the optimism of a model. The optimism corrected performance was calculated by subtracting the optimism from the apparent performance estimate, i.e. when the model was optimized and assessed for its performance on the original data set. With respect to dichotomous 60-month survival, the performance measure was the area under the ROC-curve (AUC). With respect to continuous pseudo values at 60 months and estimated survival time, the performance of the models was calculated using the root of the mean squared error (RSME).

### Variable Importance

We calculated the relative importance of each of the eight predictor variables in a model by calculating the difference between the validated performance of the full model with all eight predictor variables and the validated performance of the model with seven predictor variables, leaving out each predictor variable in turn.

### Ethics Statement

Patient data were used that had been collected prospectively and anonymously between 1981–1998. According to Dutch regulations, neither medical nor ethical approval was required to conduct the study, as no interventions were initiated and the study had no influence on medical care nor on decision making. The data was anonymised. The study was not supported financially in any way.

## Results

### Patients and Data

**Figure 1:**
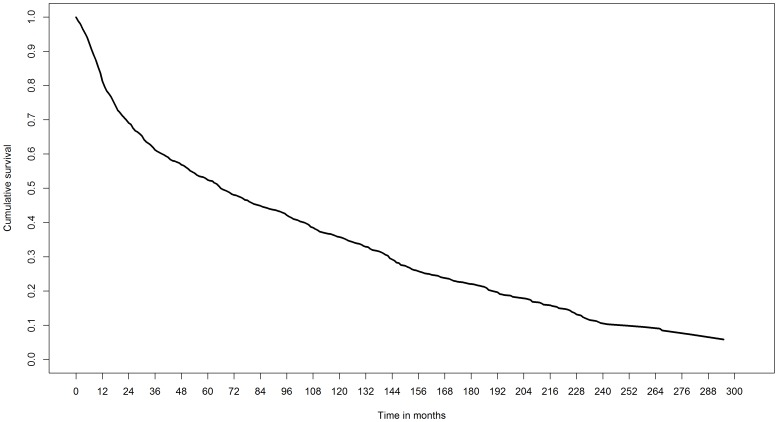
Survival pattern 1282 patients with newly diagnosed HNSCC.

**Figure 2:**
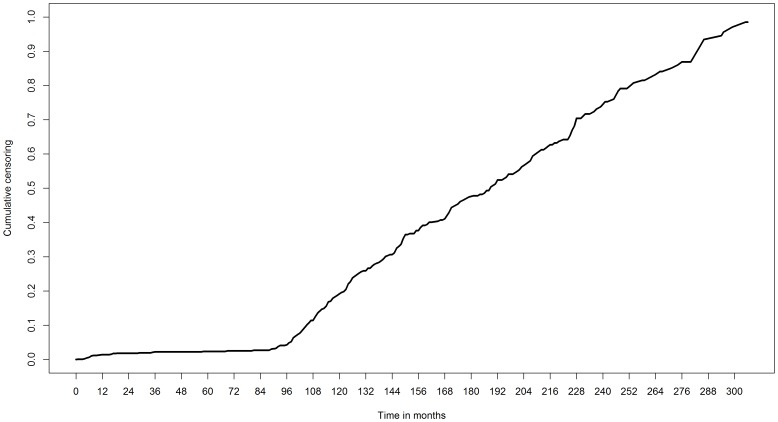
Censoring pattern 1282 patients with newly diagnosed HNSCC.

Of the 1371 patients included originally, we dropped 89 patients for whom the comorbidity was unknown. As a result, we included 1282 patients in our analysis. Of these, 986 patients died during a median follow-up of 66 months (60-month survival: 52% [95% CI: 50%−55%], Figure 1). The censoring pattern of the patients (censoring rate before 60 months: 4%) is presented in Figure 2.

Table 2 shows the overall number of events and the survival probabilities for each category of the predictor variables with respect to the Kaplan-Meier estimated 60-month survival. Several characteristics were associated with a poor 60-month survival: Tumor location in the Hypopharynx, Oral cavity and Oropharynx (60-month survival 0.33, 0.36 and 0.37 respectively), cancer stages T3, T4, and N3 (60-month survival 0.38, 0.27, 0.11 respectively), higher age (Age > = 70, 60-month survival 0.40) and severe comorbidity (Grade 3 of ACE27, 60-month survival 0.25).

**Table 2:** Overall survival and 60-month survival.

		Overall				60 months	
Variable	Value	Total (n)	Events (n)	HR	95% CI	Survival probability	95% CI
Gender	Male (ref)	1022	789	1.00	–	0.54	[0.51−0.57]
	Female	260	197	1.12	[0.96−1.31]	0.48	[0.42–0.54]
Tumor location	Glottic larynx (ref)	425	282	1.00	−	0.71	[0.67−0.75]
	Lip	85	54	0.88	[0.66−1.18]	0.75	[0.67−0.85]
	Oral cavity	261	210	2.04	[1.70−2.44]	0.36	[0.31−0.43]
	Oropharynx	148	129	2.37	[1.92−2.92]	0.37	[0.30−0.46]
	Nasopharynx	39	23	1.35	[0.88−2.06]	0.52	[0.37−0.74]
	Hypopharynx	135	123	2.83	[2.29−3.51]	0.33	[0.26−0.42]
	Supraglottic larynx	189	165	1.70	[1.40−2.06]	0.50	[0.43−0.57]
T-class	T1 (ref)	454	293	1.00	−	0.74	[0.70−0.78]
	T2	354	281	1.63	[1.38−1.92]	0.53	[0.48−0.58]
	T3	200	170	2.26	[1.87−2.73]	0.38	[0.32−0.45]
	T4	274	242	3.18	[2.68−3.78]	0.27	[0.22−0.33]
N-class	N0 (ref)	891	641	1.00	−	0.64	[0.61−0.67]
	N1	138	125	2.10	[1.73−2.54]	0.33	[0.26−0.42]
	N2	174	147	2.45	[2.04−2.94]	0.28	[0.22−0.36]
	N3	79	73	3.82	[2.99−4.89]	0.11	[0.06−0.21]
M-class	M0 (ref)	1266	972	1.00	−	0.53	[0.50−0.56]
	M1	16	14	8.51	[4.97−14.58]	0.00	−
Prior malignancies	No (ref)	1160	880	1.00	−	0.54	[0.51−0.57]
	Yes	122	106	1.62	[1.32−1.98]	0.36	[0.28−0.45]
ACE27	Grade 0 (ref)	782	574	1.00	−	0.57	[0.54−0.61]
	Grade 1	239	176	1.17	[0.99−1.39]	0.52	[0.46−0.59]
	Grade 2	185	164	1.66	[1.40−1.98]	0.44	[0.38−0.52]
	Grade 3	76	72	2.52	[1.97−3.23]	0.25	[0.17−0.37]
Age class	<50 (ref)	173	100	1.00	−	0.66	[0.59−0.74]
	50–59	339	234	1.24	[0.98−1.57]	0.59	[0.54−0.65]
	60–69	404	328	1.73	[1.38−2.16]	0.52	[0.47−0.57]
	> = 70	366	324	2.53	[2.02−3.18]	0.40	[0.36−0.46]
Total		1282	986			0.52	[0.50−0.55]

HR. Hazard ratio.

CI. Confidence interval.

### Model Performance and Optimism

We evaluated the performance of the various models with respect to the three survival related outcome variables.

For the outcome ‘dead or alive at 60 months’, the LR model had the highest optimism corrected AUC (0.791, Table 3) followed by the SVM model with linear kernel (AUC 0.787, Table 3). The NNET model performed slightly poorer (AUC 0.785, Table 3). The RPART model had the lowest AUC (0.725, Table 3).

**Table 3:** Performance of models for the outcome ‘dead or alive at 60 months’.

Dead or alive at 60 months					
Modeling technique	AUC bootstrap	AUC validated	AUC-apparent	Optimism	Optimism-corrected-AUC
LR	0.809	0.797	0.803	0.012	0.791
NNET	0.880	0.810	0.855	0.070	0.785
RPART	0.769	0.741	0.753	0.028	0.725
SVM LINEAR	0.807	0.794	0.800	0.013	0.787
SVM POLYNOMIAL	0.861	0.811	0.821	0.050	0.771
SVM RADIAL	0.872	0.813	0.825	0.059	0.766

Considering the outcome ‘pseudo values at 60 months’, the GLM model had the highest optimism corrected RMSE (0.436, Table 4). The SVM model with polynomial kernel and the NNET model performed poorly (RMSE 0.482 and 0.486 respectively, Table 4).

**Table 4:** Performance of models for the outcome ‘pseudo values at 60 months’.

Pseudo values at 60 months					
Modeling technique	RMSE bootstrap	RMSE validated	RMSE-apparent	Optimism	Optimism-corrected-RMSE
GLM	0.427	0.433	0.430	0.006	0.436
NNET	0.388	0.457	0.417	0.069	0.486
RPART	0.430	0.448	0.448	0.018	0.466
SVM LINEAR	0.461	0.470	0.460	0.009	0.469
SVM POLYNOMIAL	0.409	0.445	0.446	0.036	0.482
SVM RADIAL	0.428	0.446	0.442	0.018	0.460

Analyzing the outcome ‘estimated survival time’, the GLM model had the lowest optimism corrected RMSE (77.7, Table 5), followed by the SVM model with a linear kernel (79.2, Table 5). The NNET model had the worst RMSE (83.7, Table 5).

**Table 5:** Performance of models for the outcome ‘estimated survival time’.

Estimated survival time					
Modeling technique	RMSE bootstrap	RMSE validated	RMSE-apparent	Optimism	Optimism-corrected-RMSE
GLM	76.0	77.1	76.6	1.1	77.7
NNET	80.3	83.0	81.0	2.7	83.7
RPART	76.7	80.1	79.8	3.4	83.1
SVM LINEAR	77.4	78.7	77.9	1.3	79.2
SVM POLYNOMIAL	69.7	76.3	76.3	6.6	82.9
SVM RADIAL	69.7	76.4	76.8	6.7	83.4

The regression based models (LR and GLM) had relatively small optimism. This small optimism was also noted for the SVM models with a linear kernel. The bootstrap-estimated optimism was substantial for NNET and the more complex SVM models with polynomial and radial kernels (Table 3 to Table 5).

### Variable Importance

**Figure 3:**
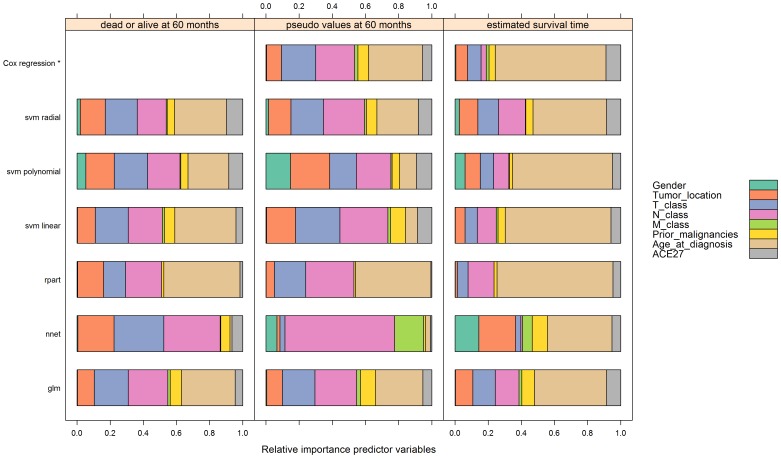
Variable importance of the models per outcome.

For each model and for each outcome we calculated the variable importance (Figure 3). We chose the parameter settings of the modeling techniques based on the highest frequency (mode) resulting from the bootstrap procedure (Table 6).

**Table 6:** Mode of the parameter settings identified as optimal in bootstrap samples.

Outcome			
Modeling technique	Dead or alive at 60 months	Pseudo values at 60 months	Estimated survival time
LR	−	−	−
NNET	size = 40	size = 30	size = 40
RPART	cp = 0.01	cp = 0.01	cp = 0.01
SVM LINEAR	cost = 0.5, gamma = 0.001	cost = 0.5, gamma = 0.001	cost = 0.5, gamma = 0.001
SVM POLYNOMIAL	cost = 50, gamma = 0.05, degree = 3	cost = 25, gamma = 0.05, degree = 3	cost = 50, gamma = 0.05, degree = 3
SVM RADIAL	cost = 50, gamma = 0.05	cost = 0.5, gamma = 0.05	cost = 50, gamma = 0.05

Figure 3 shows the variable importance for each model and for each outcome with these parameter settings.

Overall, the variables Tumor location, T-class and N-class were the most important predictor variables for predicting the dichotomous and continuous 60-months survival (Figure 3). Survival probability was considerably lower for patients with cancer stages T4 and N3 (File S3 (appendix 3), Table 7, Table 8).

**Table 7:** Logistic regression model for the outcome ‘dead or alive at 60 months’.

Logistic regression						
Variable	Value	B	SE	P-value	OR	95% CI
Tumor location	Glottic larynx (ref)	0.00	−	−	1.00	−
	Lip	0.04	0.31	0.89	1.05	[0.57−1.91]
	Oral cavity	1.00	0.21	0.00	2.73	[1.83−4.07]
	Oropharynx	0.76	0.25	0.00	2.15	[1.32−3.50]
	Nasopharynx	−0.09	0.41	0.82	0.91	[0.41−2.03]
	Hypopharynx	0.80	0.26	0.00	2.21	[1.33−3.68]
	Supraglottic larynx	0.39	0.22	0.07	1.48	[0.97−2.26]
ACE27	Grade 0 (ref)	0.00	−	−	1.00	−
	Grade 1	0.04	0.18	0.82	1.04	[0.74−1.47]
	Grade 2	0.36	0.19	0.06	1.43	[0.99−2.08]
	Grade 3	1.09	0.31	0.00	2.97	[1.62−5.45]
T-class	T1 (ref)	0.00	−	−	1.00	−
	T2	0.67	0.17	0.00	1.95	[1.38−2.74]
	T3	0.90	0.21	0.00	2.47	[1.62−3.76]
	T4	1.30	0.21	0.00	3.68	[2.44−5.55]
N-class	N0 (ref)	0.00	−	−	1.00	−
	N1	0.73	0.22	0.00	2.08	[1.34−3.22]
	N2	1.02	0.22	0.00	2.76	[1.81−4.22]
	N3	2.13	0.38	0.00	8.40	[3.98−17.72]
M-class	M0 (ref)	0.00	−	−	1.00	−
	M1	1.65	0.85	0.05	5.23	[0.99−27.63]
Prior malignancies	No (ref)	0.00	−	−	1.00	−
	Yes	1.04	0.24	0.00	2.83	[1.78−4.50]
Gender	Male (ref)	0.00	−	−	1.00	−
	Female	−0.05	0.17	0.77	0.95	[0.68−1.33]
Age at diagnosis per decade		0.49	0.06	0.00	1.63	[1.44−1.84]
Constant		−4.79	0.44	0.00	0.01	−

B: Regression coefficient.

SE: Standard error regression coefficient.

OR: Odds ratio.

CI: Confidence interval.

**Table 8:** General linear model for the outcome ‘pseudo values at 60 months’.

General linear model					
Variable	Value	B	SE	95% CI	P-value
Tumor location	Glottic larynx (ref)	0.00	−	−	−
	Lip	0.00	0.05	[−0.11−0.10]	0.93
	Oral cavity	−0.19	0.04	[−0.26–−0.11]	0.00
	Oropharynx	−0.14	0.05	[−0.23–−0.05]	0.00
	Nasopharynx	−0.06	0.08	[−0.21−0.09]	0.44
	Hypopharynx	−0.15	0.05	[−0.25–−0.06]	0.00
	Supraglottic larynx	−0.07	0.04	[−0.15−0.01]	0.08
ACE27	Grade 0 (ref)	0.00	−	−	−
	Grade 1	0.00	0.03	[−0.06−0.06]	0.99
	Grade 2	−0.07	0.04	[−0.14−0.00]	0.06
	Grade 3	−0.19	0.05	[−0.29–−0.09]	0.00
T-class	T1 (ref)	0.00	−	−	−
	T2	−0.13	0.03	[−0.20–−0.07]	0.00
	T3	−0.19	0.04	[−0.27–−0.11]	0.00
	T4	−0.27	0.04	[−0.34–−0.19]	0.00
N-class	N0 (ref)	0.00	−	−	−
	N1	−0.16	0.04	[−0.25–−0.08]	0.00
	N2	−0.22	0.04	[−0.29–−0.14]	0.00
	N3	−0.37	0.05	[−0.47–−0.26]	0.00
M-class	M0 (ref)	0.00	−	−	−
	M1	−0.27	0.11	[−0.49–−0.05]	0.02
Prior malignancies	No (ref)	0.00	−	−	−
	Yes	−0.20	0.04	[−0.28–−0.12]	0.00
Gender	Male (ref)	0.00	−	−	−
	Female	0.01	0.03	[−0.05−0.07]	0.69
Age at diagnosis per decade		−0.09	0.01	[−0.11–−0.07]	0.00
Constant		1.38	0.07	[1.24−1.52]	0.00

B: Regression coefficient.

SE: Standard error regression coefficient.

CI: Confidence interval.

For the estimated survival time, age at diagnosis was the most important predictor variable (Figure 3). Cancer stages T1 and N0 indicated a relatively good survival probability (File S3 (appendix 3), Table 9). The relative importance of each predictor variable varied substantially by the specific aspect of survival studied and modeling technique used.

**Table 9:** General linear model for the outcome ‘estimated survival time’.

General linear model					
Variable	Value	B	SE	95% CI	P-value
Tumor location	Glottic larynx (ref)	0.00	−	−	−
	Lip	−0.79	9.44	[−19.30−17.72]	0.93
	Oral cavity	−31.29	6.79	[−44.59–−17.98]	0.00
	Oropharynx	−38.62	8.26	[−54.82–−22.42]	0.00
	Nasopharynx	−21.39	13.66	[−48.17−5.38]	0.12
	Hypopharynx	−44.97	8.59	[−61.81–−28.13]	0.00
	Supraglottic larynx	−23.41	7.23	[−37.59–−9.24]	0.00
ACE27	Grade 0 (ref)	0.00	−	−	−
	Grade 1	−2.43	5.75	[−13.69−8.83]	0.67
	Grade 2	−24.39	6.41	[−36.95–−11.83]	0.00
	Grade 3	−41.36	9.37	[−59.72–−23.01]	0.00
T-class	T1 (ref)	0.00	−	−	−
	T2	−25.71	5.79	[−37.06–−14.35]	0.00
	T3	−30.65	7.18	[−44.72–−16.58]	0.00
	T4	−46.44	6.93	[−60.02–−32.86]	0.00
	N0 (ref)	0.00	−	−	−
N-class	N1	−27.65	7.60	[−42.54–−12.76]	0.00
	N2	−36.42	7.16	[−50.45–−22.40]	0.00
	N3	−56.29	9.70	[−75.29–−37.28]	0.00
M-class	M0 (ref)	0.00	−	−	−
	M1	−47.31	19.71	[−85.94–−8.68]	0.02
Prior malignancies	No (ref)	0.00	−	−	−
	Yes	−38.03	7.52	[−52.76–−23.29]	0.00
Gender	Male (ref)	0.00	−	−	−
	Female	2.99	5.56	[−7.91−13.90]	0.59
Age at diagnosis per decade		−22.71	1.88	[−26.39–−19.03]	0.00
Constant		300.47	12.58	[275.82−325.12]	0.00

B. Regression coefficient.

SE. Standard error regression coefficient.

CI. Confidence interval.

The variable plots with observed 60-month survival (dichotomous) proved to be very similar to the variable plots with pseudo values at 60 months (continuous), except for the NNET model (Figure 3).

*Cox regression was added as reference technique.

## Discussion

In this study, we demonstrated that pseudo values as described by Klein et al. [10], [11] enable statistically appropriate analyses of survival outcomes when used in in three variants of support vector machines (SVM), neural networks (NNET), general linear models (GLM), recursive partitioning (RPART) and logistic regression (LR). We showed that pseudo values enabled us to apply these techniques to predict survival in a case study of 1282 Dutch patients with newly diagnosed HNSCC, and to compare the performance of the resulting models.

Our analysis showed that conventional regression analysis approaches (logistic regression and the generalized linear model) outperformed the performance of relatively modern modeling techniques. However, the SVM model with an optimal setting and a linear kernel performed only slightly worse with respect to our outcomes. The NNET model and the RPART model performed relatively poorly.

We compared the performance of the alternative modeling techniques in predicting three variants of survival outcome for our case study. The first, admittedly rather simplistic, outcome variable was based on the 60-month survival in terms of dead or alive. This outcome may produce bias unless the censoring rate is small (4% in our study). The other two outcome variables were defined by means of pseudo values, which were derived from the Kaplan Meier survival function.

A drawback of outcome definitions for 60 months is that they only consider survival at a particular point in time rather than the full survival curve. By contrast, the approach with the estimated survival time is attractive, because it considers the full survival curve. We consider the total expected survival time the most relevant to inform patients about their prognosis and to support decision making.

In our study, SVM models with a linear kernel and optimal settings performed slightly worse than conventional regression modeling. These findings are in line with other studies that used support vector machines for analyzing survival [3]–[6]. On the other hand, our findings also support the results of previous studies that relied on Cox regression modeling to predict the five year mortality and the overall mortality of newly diagnosed patients with HNSCC [15]–[17].

None of the investigated models showed a very satisfactory performance. This may possibly be explained by the low signal-to-noise ratio in our data. In 1998, Ennis et al. discussed the predictive performance of adaptive non-linear algorithms versus conventional statistical techniques. Based on their quite negative findings for the more modern algorithms, they postulated that adaptive non-linear methods may be most useful in problems with high signal-to-noise ratios, which sometimes occur in engineering and physical science. Since the signal-to-noise ratio is often quite low in medical prediction studies, they concluded that modern methods may have less to offer [24].

A limitation of this study is that the results were based on a single cohort of 1282 Dutch patients, diagnosed at a single center [16]. We had to rely on bootstrap validation to estimate the performance of alternative modeling techniques. On the other hand, the number of events was more than sufficient to allow for detailed statistical modeling with modern techniques for the relatively small set of candidate predictors.

We showed that the use of pseudo values opens new possibilities for analyzing survival problems with techniques other than conventional regression techniques. The validity of the pseudo value approach is supported by the concordance between Cox regression modeling for censored survival time and Generalized Estimating Equation modeling (GEE) using a log-minus-log link function [11]. Therefore, this approach deserves a central role in the ongoing search for improved prediction models for survival. On the other hand, our results also show that it may be hard to find modeling approaches that are superior to conventional regression analysis in terms of performance, applicability and simplicity.

In conclusion, the use of pseudo values makes it readily possible to analyze survival time with alternative modeling techniques, to compare their performance and to search further for promising alternative modeling techniques to analyze survival time. In our case study on patients with newly diagnosed HNSCC, none of the alternative modeling techniques provided better predictions for survival than conventional regression modeling techniques. The estimated importance of predictors depends on the specific aspect of survival studied and the modeling technique used.

## Supporting Information

File S1 Appendix 1.
(DOCX)

File S2 Appendix 2.
(DOCX)

File S3 Appendix 3.
(DOCX)
