# Supplementary material for: Prediction of Survival with Alternative Modeling Techniques Using Pseudo Values
Source: PLoS One. 2014 Jun 20;9(6):e100234. doi: 10.1371/journal.pone.0100234 (PMC4065009; doi:10.1371/journal.pone.0100234)
Supplement: File S1 — Appendix 1. (DOCX) [file pone.0100234.s001.docx]

**APPENDIX 1**

This appendix describes the calculation of the pseudo values, the calculation of the estimated survival time and the interpretation of the pseudo values [10].

**Calculation of the pseudo values and the estimated survival time**

Let S(t) be the estimated Kaplan-Meier survival function. We calculated the pseudo values J_i_(t) for the i^th^ patient as J_i_(t)=nS(t)-(n-1)S^(-i)^(t) with S^(-i)^(t) the survival function without the i^th^ patient.

J_i_(t) can be considered as the individual survival function for the i^th^ patient. The area under the survival curve of J_i_(t) is the survival time for the i^th^ patient.

In our study, we calculated the pseudo values at the time points t=12, 24, …, 300. For the i^th^ patient, we calculated the estimated survival time (EST_i_) as EST_i_=12(J_i_(12)+J_i_(24)+…+J_i_(300)).

**Examples**

The following two examples are meant as an illustration how to interpret the pseudo values of a patient. We do not consider the case of negative pseudo values.

***Example 1 (censored case):***

Suppose a patient has the following series of pseudo values at the particular time points:

The interpretation of the series of pseudo values for this patient is:

The patient did not die because all pseudo values are positive. The follow-up time point lies between the time points 204 and 216 because the increasing pattern of the pseudo values changes into a decreasing pattern.

***Example 2 (uncensored case):***

Suppose a patient has the following series of pseudo values at the particular time points:

The interpretation of the series of pseudo values for this patient is:

The patient died between the time points 108 and 120 because the pseudo values change from a positive value into a negative value.

Figure 4 shows that the Kaplan-Meier survival curve nearly matches the curve of the mean pseudo values at each time point.

Figure 5 shows that the sum of the pseudo values multiplied by 12 can be considered as an estimation of the area under the survival curve and therefore as an estimation of the expected survival time for a patient.

For the modeling of the various models we therefore used this variable as outcome variable to estimate the survival time of a patient.
