# Supplementary material for: Prediction of Survival with Alternative Modeling Techniques Using Pseudo Values
Source: PLoS One. 2014 Jun 20;9(6):e100234. doi: 10.1371/journal.pone.0100234 (PMC4065009; doi:10.1371/journal.pone.0100234)
Supplement: File S2 — Appendix 2. (DOCX) [file pone.0100234.s002.docx]

**APPENDIX 2**

This appendix contains a description of the evaluated modeling techniques and their parameters, based on literature and articles of various authors [20-24]. The standard regression techniques logistic regression (LR) and generalized linear model (GLM) are assumed to be familiar.

**Description of the models**

Support vector machines

A Support Vector Machine (SVM) performs classification tasks by constructing hyperplanes with a margin in a multidimensional space that separates cases from different classes. SVM can efficiently perform a non-linear classification or regression task using different kernels (radial, linear and polynomial). The tuning parameters for SVM are the C-parameter (cost), which regulates the margin width, and the gamma-parameter for the kernel calculation. SVM claims to be a robust classification and regression technique that maximizes the predictive accuracy of a model without overfitting the training data. SVM may particularly be suited to analyze data with large numbers of predictor variables.

Neural net

A neural network (NNET), sometimes called a multilayer perceptron, works by simulating a large number of interconnected simple processing units, which are arranged in layers. There are three parts in a neural network: an input layer, with units representing the predictor variables, one or more hidden layers and an output layer, with a unit representing the outcome variable. The units are connected with varying connection strengths or weights. Input data are presented to the input layer and values are propagated from there to the next layer. Then, a prediction is delivered from the output layer. The network learns by examining individual records, generating a prediction for each record and making adjustments to the weights whenever it makes an incorrect prediction. This process is repeated many times, and the network continues to improve its predictions until one or more of the stopping criteria have been met. Initially, all weights are random, and the predictions that come out of the net are nonsensical. The network learns through training. Records for which the output is known are repeatedly presented to the network, and the predictions it gives are compared to the known outcomes. As training progresses, the network becomes increasingly accurate in replicating the known outcomes. Once trained, the network can be applied to new patients for whom the outcome is unknown. The parameters of NNET are the size-parameter (number of units in the layer) and decay-parameter.

Recursive partitioning

Recursive partitioning (RPART) is a tree-based classification and prediction modelling technique which uses recursive partitioning to split the training records into segments with similar output variable values. The modeling starts by examining the input variables to find the best split, measured by the reduction in an impurity index that results from the split. The split defines two subgroups, each of which is subsequently split into two further subgroups and so on, until the stopping criterion is met. The parameter of RPART is the cp-parameter (cost complexity factor). A cp-value of 0.001 for example regulates that a split must decrease the overall lack of fit by a factor of 0.001.
