# Supplementary material for: Prediction of Survival with Alternative Modeling Techniques Using Pseudo Values
Source: PLoS One. 2014 Jun 20;9(6):e100234. doi: 10.1371/journal.pone.0100234 (PMC4065009; doi:10.1371/journal.pone.0100234)
Supplement: File S3 — Appendix 3. (DOCX) [file pone.0100234.s003.docx]

**APPENDIX 3**

This appendix contains the coefficients of the regression models.

**Coefficients regression models**

<Table 7 Logistic regression model outcome ‘dead or alive at 60 months’>

<Table 8 General linear model outcome ‘pseudo values at 60 months’>

<Table 9 General linear model outcome ‘estimated survival time’>
